# Angiotensin II Receptor Blocker Attenuates Intrarenal Renin-Angiotensin-System and Podocyte Injury in Rats with Myocardial Infarction

**DOI:** 10.1371/journal.pone.0067242

**Published:** 2013-06-14

**Authors:** Zhu-zhi Wen, Mu-yan Cai, Zun Mai, Dong-mei Jin, Yang-xin Chen, Hui Huang, Deng-feng Geng, Jing-feng Wang

**Affiliations:** 1 Department of Cardiology, Sun Yat-sen Memorial Hospital, Sun Yat-sen University, Guangzhou, China; 2 Guandong Province Key Laboratory of Arrhythmia and Electrophysiology, Sun Yat-sen Memorial Hospital, Sun Yat-sen University, Guangzhou, China; 3 Department of Pathology, Cancer Center, Sun Yat-sen University, Guangzhou, China; 4 Breast Tumor Center, Sun Yat-sen Memorial Hospital, Sun Yat-sen University, Guangzhou, China; 5 Department of Rehabilitation Medicine, Sun Yat-sen Memorial Hospital, Sun Yat-sen University, Guangzhou, China; Virginia Commonwealth University, United States of America

## Abstract

The mechanisms and mediators underlying common renal impairment after myocardial infarction (MI) are still poorly understood. The present study aimed to test the hypothesis that angiotensin II type 1 receptor blockers (ARBs) provides renoprotective effects after MI by preventing augmented intrarenal renin-angiotensin-system (RAS)-induced podocyte injury. Sprague–Dawley rats that underwent ligation of their coronary arteries were treated with losartan (20 mg/kg/d) or vehicle for 3 or 9 weeks. Renal function, histology and molecular changes were assessed. The current study revealed that MI-induced glomerular podocyte injury was identified by increased immunostaining for desmin and p16^ink4a^, decreased immunostaining for Wilms’ tumor-1 and podocin mRNA expression, and an induced increase of blood cystatin C at both 3 and 9 weeks. These changes were associated with increased intrarenal angiotensin II levels and enhanced expressions of angiotensinogen mRNA and angiotensin II receptor mRNA and protein. These changes were also associated with decreased levels of insulin-like growth factor (IGF-1) and decreased expressions of IGF-1 receptor (IGF-1R) protein and mRNA and phosphorylated(*p*)-Akt protein at 9 weeks, as well as increased expressions of 8-hydroxy-2’-deoxyguanosine at both time points. Treatment with losartan significantly attenuated desmin- and p16^ink4a^-positive podocytes, restored podocin mRNA expression, and decreased blood cystatin C levels. Losartan also prevented RAS activation and oxidative stress and restored the IGF-1/IGF-1R/Akt pathway. In conclusion, ARBs prevent the progression of renal impairment after MI via podocyte protection, partially by inhibiting the activation of the local RAS with subsequent enhanced oxidative stress and an inhibited IGF-1/IGF-1R/Akt pathway.

## Introduction

Myocardial infarction (MI) with resultant chronic heart failure (CHF) is a leading cause of mortality and morbidity in developed countries. Accelerated and progressive decline in renal function is common and associated with increased mortality in MI patients during hospitalization [1,2]. Renal parenchymal damage is closely associated with deteriorating renal function following MI [3,4]. Therefore, it is of paramount importance to explore potential mechanisms involved in renal parenchymal damage in order to prevent the onset and progression of renal dysfunction after the initiation of MI with subsequent CHF.

However, mechanisms underlying renal parenchymal impairment following MI are not yet fully clear. Growing evidence has suggested that undue activation of the local renin-angiotensin-system (RAS) within the kidneys in an inappropriate fashion is an important contributor to the pathogenesis of renal injury. MI will induce a significant increase in RAS components, such as increased renal levels of renin mRNA, angiotensinogen (AGT) mRNA and angiotensin II (AngII) concentration [5], and up-regulated expressions of AngII receptors [6]. Our previous study [6] revealed that MI-induced the local up-regulation of AngII type 1 receptor (AT1R) in the kidneys was associated with an increase in serum cystatin C levels which has been reported to be coincided with histopathologic intrarenal damage and augmented AngII in rats with acute kidney injury [7]. In addition, the detrimental effects of enhanced intrarenal RAS on the pathogenesis of progressive renal impairment after heart failure may be attenuated by the intervention with RAS inhibitions [8-11].

Podocytes, as key parenchymal cells within the glomerular and modulators to maintain the glomerular filtration barrier, have been found to be potentially injured by the activation of the intrarenal RAS in rat models with non-MI heart failure, resulting in the leakage of urine protein [11]. Podocytes express the AT1Rs, and could thus be injured by enhanced AngII, leading to proteinuria, glomerulosclerosis and even end stage kidney disease [12-14]. Administration of AT1R blockers (ARBs) could prevent the initiation and deterioration of podocyte injury [11,13]. Further findings [11,15] also support that ARB outweighing other drugs, such as nifedipine, atenolol and hydralazine, should be preferentially chosen to attenuate local RAS-induced podocyte injury, providing new insight into the treatment of cardiorenal syndrome.

Our previous findings suggest that serum cystatin C rather than blood creatinine and urine protein is a better early marker for renal parenchymal damage post-MI heart failure [6]. However, few studies have directly explored the role of podocyte injury in serum cystatin C changes. Therefore, the authors speculated that MI could trigger activation of the intrarenal RAS axis, contributing to podocyte injury with resultant increased serum cystatin C levels. The present study aimed to determine the changes in the local renal RAS components and glomerular podocytes of rats with post-MI heart failure, with or without ARB treatment, and to explore potential mechanisms involved in the processes of these changes.

## Materials and Methods

### Animals and experimental myocardial infarction

Experimental MI in rats were induced by left coronary artery ligation by a method widely described. Briefly, male Sprague-Dawley rats weighing 280-310 g were anesthetized with ketamine/xylazine (50/5 mg/kg, i.p.). Rats were intubated and then ventilated with a rodent respirator. A left thoracotomy was performed via the left fourth intercostal space and the heart was exposed. After the pericardium was opened, the left anterior descending coronary artery was ligated with a 6-0 silk suture. The chest was then closed with a soft tube in the cavity in order to withdraw air or blood. After ventilation with the room air for approximately 5 min, the animal was gradually weaned from the respirator once spontaneous respiration resumed, and it remained supervised until completely conscious. Sham-operated animals received the same surgical procedures with the exception of the ligations.

### Ethics Statement

Animals used in these experiments were treated in accordance with the *Guide for the Care and Use of Laboratory Animals* (NIH Publication No. 85-23, revised 1996), and the study protocols were approved by the Animal Ethics Committee of Sun Yat-sen University.

### Experimental groups and treatment

Forty-eight hours after surgery, survival MI animals were randomly allocated to be treated with losartan (20 mg/kg/d) or vehicle. The determination of losartan dose was based on our preliminary studies which revealed that losartan at this dose improved cardiac function better than at the dose of 10 mg/kg/d and had less side effect, such as hypotension, than at the dose of 30 mg/kg/d in rats post-MI (data not shown). This dose of losartan has also been demonstrated to play positive effect in improving cardiac function and attenuating cardiac hypertrophy without symptoms of side effect in rats post-MI [16]. Male rats in this study were randomly divided into 4 groups: the normal group (n=8 for both 3 and 9 weeks), the sham-operated (sham) group (n=8 and 10 for 3 and 9 weeks, respectively), the MI group (n=14 and 10 for 3 and 9 weeks, respectively), and the MI plus losartan (MI+ los) group (n= 20 and 18 for 3 and 9 weeks, respectively). Losartan treatment was initiated 2 days post-MI. Drug administration was performed via gastric gavage daily for 3 or 9 weeks. Double distilled water was also given as a vehicle by gastric gavage to rats without the losartan treatment to avoid the possible physiological alterations associated with gavage-induced stress.

### Sample collection

Trunk blood was collected and the supernatants were stored at −80°C for further analysis. After a 24-h acclimatization period, the supernatants of the urine samples collected from metabolic cages 2 days prior to sacrifice at each time point were stored at -20°C for urinary total protein analysis.

Immediately after euthanasia, induced with an overdose of sodium pentobarbital (100 mg/kg, i.p.), approximately 100 mg of left renal cortical tissue was homogenized in 0.1 mol/l acetic acid (10%, wt/vol) containing EDTA and protease inhibitors. The supernatants were obtained after centrifugation. Another 50 mg of left renal cortical tissue was homogenized in cold PBS and supernatants were obtained for the measurement of insulin-like growth factor-1 (IGF-1). The remaining left kidney tissue was cut and fixed in 4% formalin and embedded in paraffin. The right renal tissues were snap-frozen in liquid nitrogen and then stored at −80°C for RNA and protein measurement.

### Histological and immunohistochemical examinations

Kidney sections (4-μm) were processed for immunohistochemical staining according to the previously described protocol [17]. The slides were de-paraffinized in xylene, rehydrated through graded alcohol, immersed in 3% hydrogen peroxide to block endogenous peroxidase activity, and antigen-retrieved by pressure cooking in citrate buffer (pH=6). After nonspecific binding blocking, the slides were incubated with Wilms’ tumor-1 (WT-1) (Santa Cruz, CA), anti-desmin (Dako, Denmark), p16^ink4a^ (Santa Cruz, CA) or anti-8-hydroxy-2'-deoxyguanosine (8-OHdG) (Japan Institute for the control of Aging, Japan) and stored overnight at 4°C. The slides were sequentially incubated with a secondary antibody (Dako, Denmark) and stained with 3,3-diaminobenzidine (DAB). Finally, the sections were counterstained with Mayer’s hematoxylin, dehydrated, and mounted. A negative control was obtained by replacing the primary antibody with a normal murine or rabbit IgG. In each glomerulus, the percentage of desmin-positive area within the glomerular area and the number of WT-1, p16^ink4a^ and 8-OHdG-positive podocytes per glomerulus were counted using Image-Pro Plus software (Media Cybernetics, MD). A total of 20 consecutive glomeruli per section were examined by a pathological expert in a blind manner.

### Reverse transcription polymerase chain reaction

Reverse transcriptase-polymerase chain reaction (RT-PCR) was used to determine changes in renal expressions of AT1R, AT2R, rennin, AGT, podocin and IGF-1 receptor (IGF-1R) mRNA. Total RNA was isolated from renal cortical tissue with Trizol reagent (GIBCO Invitrogen), and the amount of RNA was measured with spectrophotometry. Equal RNA was used for cDNA synthesis with moloney murine leukemia virus and similarly reverse transcribed with primers, and primers for the β-actin were also included in each reaction as an internal control (Table 1). RT was performed at 42°C for 1 h, followed by a denaturation at 94°C for 5 min. The cycle profile of PCR was performed as follows: denaturation for 30 sec at 94°C, annealing for 30 sec at 60°C (AT1R: 58°C; renin: 57°C; AGT: 58°C; β-actin: 57°C), and extension for 45 sec at 72°C. Different numbers of cycles for amplification were performed. The amplification products of each PCR reaction were separated using 2% agarose gel electrophoresis. After quantifying band intensities by densitometry, the relative steady-state level of mRNA was calculated after normalizing to β-actin.

**Table 1:** PCR primer sequences and conditions.

Genes	Sequence	Cycles	Length,bp
AT-1R		26	*173*
Sense	5'- GACCATTCACCCTGCCTCAG- 3'		
Anti-sense	5'- CCAGACCCACCAATCCATCC-3'		
AT-2R		28	156
Sense	5'-GGCAGATAAGCATTTGGAAGC-3'		
Anti-sense	5'-AAGTCAGCCACAGCCAGATTG-3'		
Renin		26	112
Sense	5'-GGTGCTAAAGGAGGAAGTGTTT-3'		
Anti-sense	5'-GTGAAAGTTGCCCTGGTAATG-3'		
Angiotensinogen		25	244
Sense	5’-TTGGGTGCTGAGGCAAATCT-3’		
Anti-sense	5’-CCACATTTTGGGGGTTATCC-3’		
Podocin		25	144
Sense	5'-AGTGCGGGTGATTGCTGC-3'		
Anti-sense	5'-GTGGACGGCTTGTCTGTG-3'		
IGF-1R		25	101
Sense	5’-CGAGCAAGTTCTTCGTTTCGT-3’		
Anti-sense	5’-TGTACTGCCAGCACATGCG-3’		
β-actin		20	229
Sense	5'-CGTAAAGACCTCTATGCCAACA-3'		
Anti-sense	5'-CGGACTCATCGTACTCCTGCT-3'		

### Western Blotting

Total protein was extracted from renal cortical tissue with modified radioimmunoprecipitation assay (RIPA) buffer. Equal protein lysates were separated by 10% polyacrylamide gel, transferred to polyvinylidene difluoride membrane and then subjected to immunoblotting with antibodies anti-AT1R (Abcam, UK), anti-AT2R (Abcam, UK), anti-IGF-1R (Abcam, UK), anti-phosphorylated(*p*)-Akt (CST, USA), anti-Akt (CST, USA) and anti-GAPDH (CST, USA) at 4°C overnight. After washing, the membranes were incubated with horseradish peroxidase–conjugated secondary antibodies, and visualized using the ECL chemiluminescence system. The densitometry analysis was performed using the Bio-Rad image detection system and Quantity One software (Bio-Rad, CA, USA). After quantifying band intensities by densitometry, the relative steady-state level of protein was calculated after normalizing to GAPDH.

### Enzyme-linked immunosorbent assay

Samples of tissue homogenate supernatant and blood serum collected at 3 and 9 weeks were tested for AngII, IGF-1 and cystatin C contents. Enzyme-linked immunosorbent assays were performed using commercial kits against AngII (SPI-BIO, France), IGF-1 (MG100, R&D Systems) and cystatin C (MSCTC0, R&D Systems) according to the manufacturer’s instructions.

### Other parameters

Echocardiographic measurements were performed 3 and 9 weeks after treatment using a high-resolution echocardiographic imaging system equipped with a 16 MHz transducer (Vevo2100, Visualsonics, Canada). Systolic blood pressure (SBP), diastolic blood pressure (DBP) and heart rate during the 3- and 9-week treatment periods were measured five consecutive times in conscious rats using a tail-cuff plethysmograph (model BP-98A; Softron Co., Japan). Serum creatinine, blood urea nitrogen (BUN) and glucose, as well as urinary concentration of total protein, were detected using commercial kits (Beijing Leadman Biochemistry Technology Co. Ltd, China) according to the manufacturer’s instructions.

### Statistical Analysis

All quantitative data are presented as the mean ± SEM. According to normality test results, data were compared using a one-way ANOVA followed by a LSD *post hoc* test. Two-tailed *P*-values < 0.05 were considered significant and adjusted *P*-values were used among the subgroup comparison analyses. All statistical analyses were performed with the software package SPSS 16.0 (IBM, USA) for Windows.

## Results

### General characteristics

When compared to the normal or sham-operated animals, animals that underwent a ligation of the coronary artery had significantly decreased heart functions at both time points (Table 2). Administration of losartan improved ejection fraction at the end of both time points, but significance was revealed only at 9 weeks. Animals in the MI-induced group showed insignificant changes in the SBP and DBP, whereas losartan significantly reduced SBP and DBP readings at both time points. Renal weights and ratios of renal weight to body weight did not differ from each other at both time points.

**Table 2:** General characteristics and biological parameters.

	Normal	Sham	MI	MI+los	*P*
3 week (n)	8	8	14	20	
Body weight (g)	281.0±4.10	297.2±8.84	318.2±6.95 ^a^	332.4±7.92 ^a^	<0.001
Renal weight (g)	1.92±0.04	1.92±0.05	2.00±0.06	2.10±0.08	0.261
Renal weight/body weight (*10^-3^)	6.84±0.19	6.49±0.27	6.30±0.12	6.30±0.14	0.149
Heart rate (beat/min)	353±22	363±23	359±8	384±12	0.324
Systolic blood pressure (mmHg)	127±5	134±4	120±2	100±3 ^abc^	<0.001
Diastolic blood pressure (mmHg)	97±4	96±4	91±2	78±3 ^abc^	<0.001
Ejection fraction (%)	61.9±0.85	63.9±2.01	28.5±1.44 ^ab^	35.2±2.59 ^ab^	<0.001
Blood glucose (mmol/L)	6.2±0.46	6.2±0.64	4.6±0.08	5.8±0.22	0.021
9 week (n)	8	10	10	18	
Body weight (g)	346.7±6.15	348.3±6.77	349.8±11.98	339.3±3.77	0.720
Renal weight (g)	1.89±0.04	1.88±0.01	2.06±0.05	1.89±0.05	0.093
Renal weight/body weight (*10^-3^)	5.48±0.21	5.40±0.11	5.91±0.18	5.56±0.13	0.226
Heart rate (beat/min)	339±10	332±17	337±10	329±9	0.943
Systolic blood pressure (mmHg)	126±1	123±3	125±4	97±3 ^abc^	<0.001
Diastolic blood pressure (mmHg)	96±1	94±1	96±2	77±2 ^abc^	<0.001
Ejection fraction (%)	60.6±0.62	59.8±1.30	31.0±3.11 ^ab^	39.1±1.77 ^abc^	<0.001
Blood glucose (mmol/L)	6.5±0.25	6.4±0.32	5.5±0.22	5.8±0.35	0.250

Data are presented as means ± SEM. *P*-value based on one-way ANOVA followed by a LSD test. ^a^ *P* < 0.008 vs. normal group; ^b^ *P* < 0.008 vs. sham group; ^c^ *P* < 0.008 vs. MI alone. MI, myocardial infarction; los, losartan.

### Changes of local RAS components

**Figure 1:**
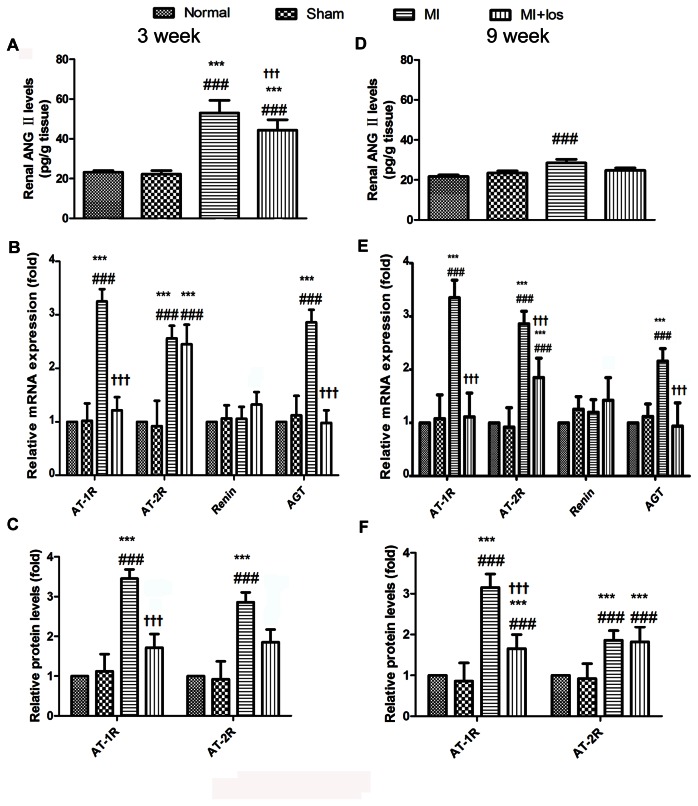
Local changes in the renin-angiotensin-system (RAS) components in normal, sham, MI and MI+los rats. (A) Renal AngII contents at 3 weeks. (B) Levels of AT1R, AT2R, renin and AGT mRNA at 3 weeks. (C) Expressions of AT1R and AT2R proteins at 3 weeks. (D) Renal AngII contents at 9 weeks. (E) Levels of AT1R, AT2R, renin and AGT mRNA at 9 weeks. (F) Expressions of AT1R and AT2R proteins at 9 weeks. Data are shown as the mean ± SEM. ###*P* < 0.008 vs. normal; ***P < 0.008 vs. sham; ††† *P* < 0.008 vs. MI. MI, myocardial infarction; los, losartan; AngII, angiotensin II; AT1/2R, angiotensin II type 1/2 receptor; AGT, angiotensinogen.

MI-induced animals had higher levels of AngII in the renal cortex than normal or sham animals at 3 and 9 weeks (Figure 1). The MI group also had higher levels of both AT1R and AT2R in the expressions of mRNA and protein than the normal or sham groups at 3 and 9 weeks (Figure 1). Additionally, MI-induced animals showed increased levels of AGT but not renin by RT-PCR in renal cortical tissue. Treatment with losartan prevented the augmentation of kidney AngII levels as well as the expressions of AT1R mRNA and protein. Increased expressions of AGT but not renin mRNA at both therapy periods could also be significantly inhibited by losartan (Figure 1B, 1E).

### Changes in renal function

In order to make an easy explanation, samples of new set of experimental MI animals, as well as samples of our previous study [6] were used to assess renal function again, which may overlap with that in our previous article. Just as our previous findings [6], cystatin C, a sensitive marker of early renal impairment, was revealed to significantly increase in animals after MI, which was higher than those in normal or sham animals (Table **S1**). ARB treatment significantly decreased serum concentration of cystatin C both at 3 and 9 weeks. The changes of levels in serum creatinine, BUN and urine protein were just the same as those reported in our previous study [6]. For example, MI-induced group showed enhanced levels of BUN at the end of 3 but not 9 weeks and was not affected by the treatment of losartan. Additionally, no significant changes in serum creatinine and urine protein were demonstrated among the four animal groups at both time points.

### Renal histological and glomerular podocyte changes

**Figure 2:**
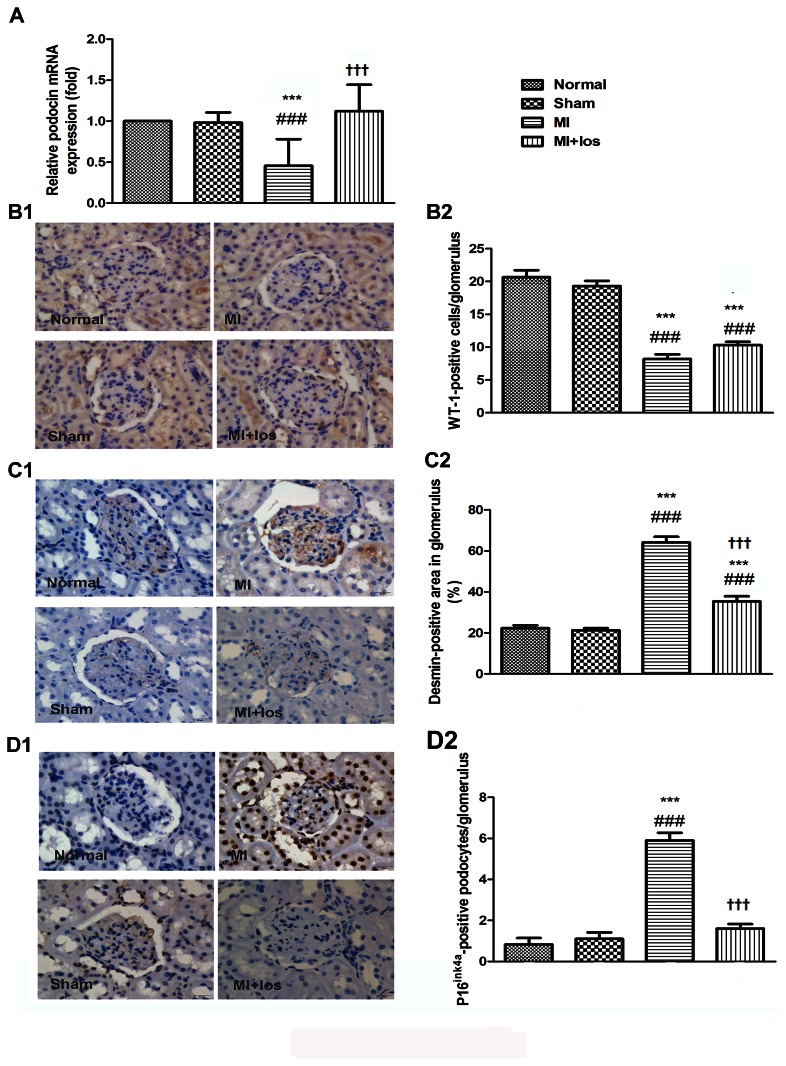
Representative podocyte injury in normal, sham, MI and MI+los rats at 3 weeks. (A) Podocyte injury revealed by mRNA expression of glomerular podocin (B1) Representative immunohistochemical staining for WT-1 (original magnification, ×400) (B2) The average number of WT-1-positive podocytes per glomerulus (C1) Representative immunohistochemical staining for desmin (original magnification, ×400) (C2) The relative desmin-stained area in the glomerulus as a percentage of total glomerular area (D1) Representative immunohistochemical staining for p16^ink4a^ (original magnification, ×400) (D2) The average number of p16^ink4a^-positive podocytes per glomerulus. Data are presented as the mean ± SEM. ###*P* < 0.008 vs. normal; ***P < 0.008 vs. sham; ††† *P* < 0.008 vs. MI. MI, myocardial infarction; los, losartan; WT-1, Wilms’ tumor-1.

**Figure 3:**
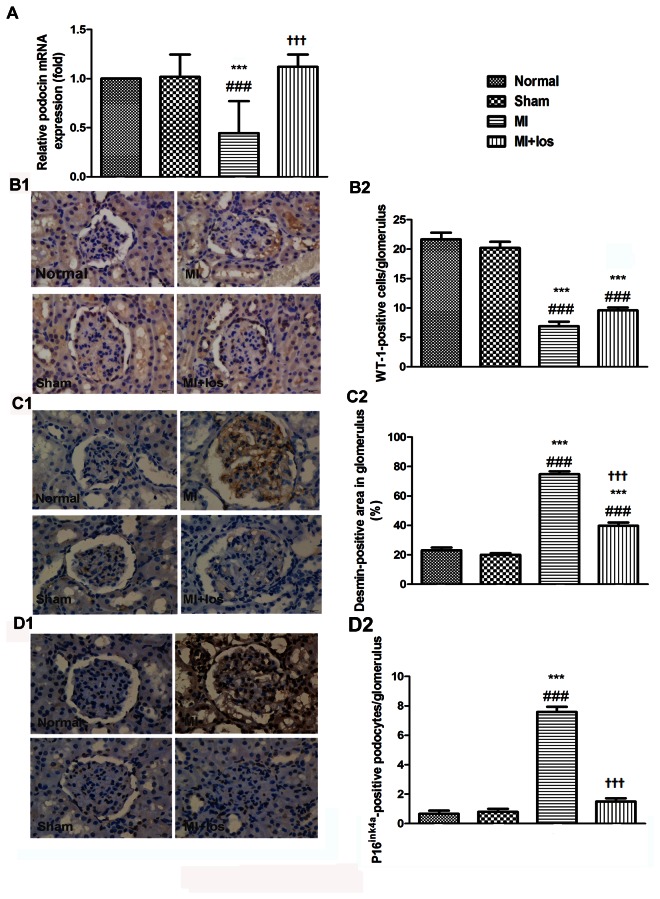
Representative podocyte injury in normal, sham, MI and MI+los rats at 9 weeks. (A) Podocyte injury revealed by mRNA expression of glomerular podocin (B1) Representative immunohistochemical staining for WT-1 (original magnification, ×400) (B2) The average number of WT-1-positive podocytes per glomerulus (C1) Representative immunohistochemical staining for desmin (original magnification, ×400) (C2) The relative desmin-stained area in the glomerulus as a percentage of total glomerular area (D1) Representative immunohistochemical staining for p16^ink4a^ (original magnification, ×400) (D2) The average number of p16^ink4a^-positive podocytes per glomerulus. Data are shown as the mean ± SEM. ###*P* < 0.008 vs. normal; ***P < 0.008 vs. sham; ††† *P* < 0.008 vs. MI. MI, myocardial infarction; los, losartan; WT-1, Wilms’ tumor-1.

As compared with the normal or sham group, the kidneys in the MI-induced group had more injured podocytes as identified by desmin-positive immunostaining and decreased podocyte numbers as identified by WT-1-positive immunostaining in the glomerulus. Consistently, the mRNA expression of podocin indicating normal function of the slit diaphragm between podocytes was revealed to be down-regulated in MI animals compared to normal or sham animals. Furthermore, the number of senescent podocytes as tested by the p16^ink4a^ assay in the glomerulus was significantly higher in MI-induced animals than in animals without operation or with a sham operation. However, treatment with losartan significantly attenuated desmin- and p16^ink4a^-positive podocytes and restored podocin mRNA expression at the end of both periods. Additionally, when compared to the MI-induced animals alone, MI animals treated with losartan had more WT-1-positive podocytes at 3 (*P* = 0.042) and 9 (*P* = 0.025) weeks. All these changes are shown in the Figure **2** and Figure **3**.

### Potential mechanisms involved in MI-induced renal impairment

**Figure 4:**
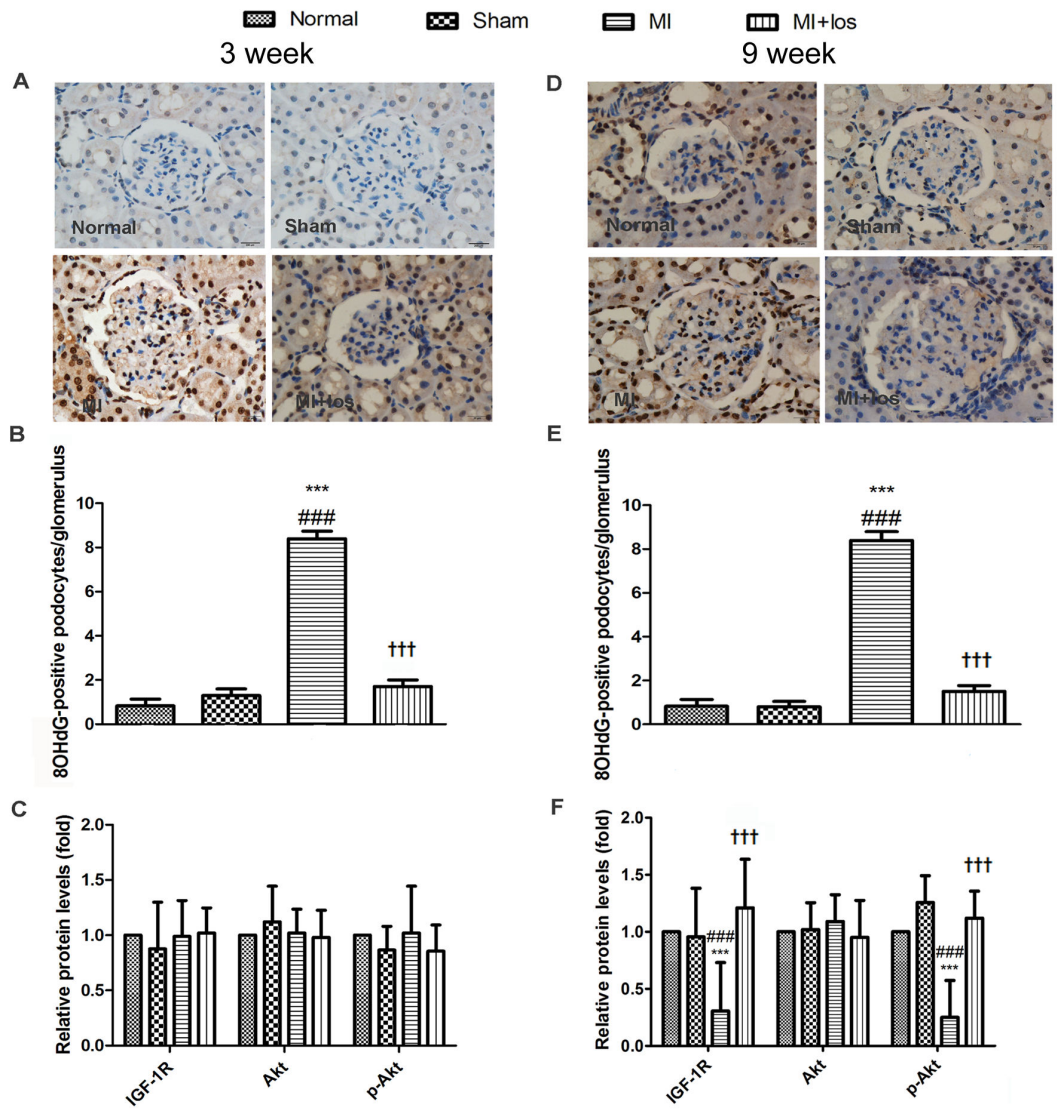
Potential mechanisms involved in podocyte injury in normal, sham, MI and MI+los rats. (A) Representative immunohistochemical staining for 8-OHdG (original magnification, ×400) at 3 weeks. (B) The average number of 8-OHdG-positive podocytes per glomerulus at 3 weeks. (C) Expressions of IGF-1R, Akt and phosphorylated(p)-Akt proteins at 3 weeks. (D) Representative immunohistochemical staining for 8-OHdG (original magnification, ×400) at 9 weeks. (E) The average number of 8-OHdG-positive podocytes per glomerulus at 9 weeks. (F) Expressions of IGF-1R, Akt and phosphorylated(p)-Akt proteins at 9 weeks. Data are presented as the mean ± SEM. ###*P* < 0.008 vs. normal; ***P < 0.008 vs. sham; ††† *P* < 0.008 vs. MI. MI, myocardial infarction; los, losartan; 8-OhdG, 8-hydroxy-2'-deoxyguanosine; IGF-1(R), insulin-like growth factor-1(receptor).

**Figure 5:**
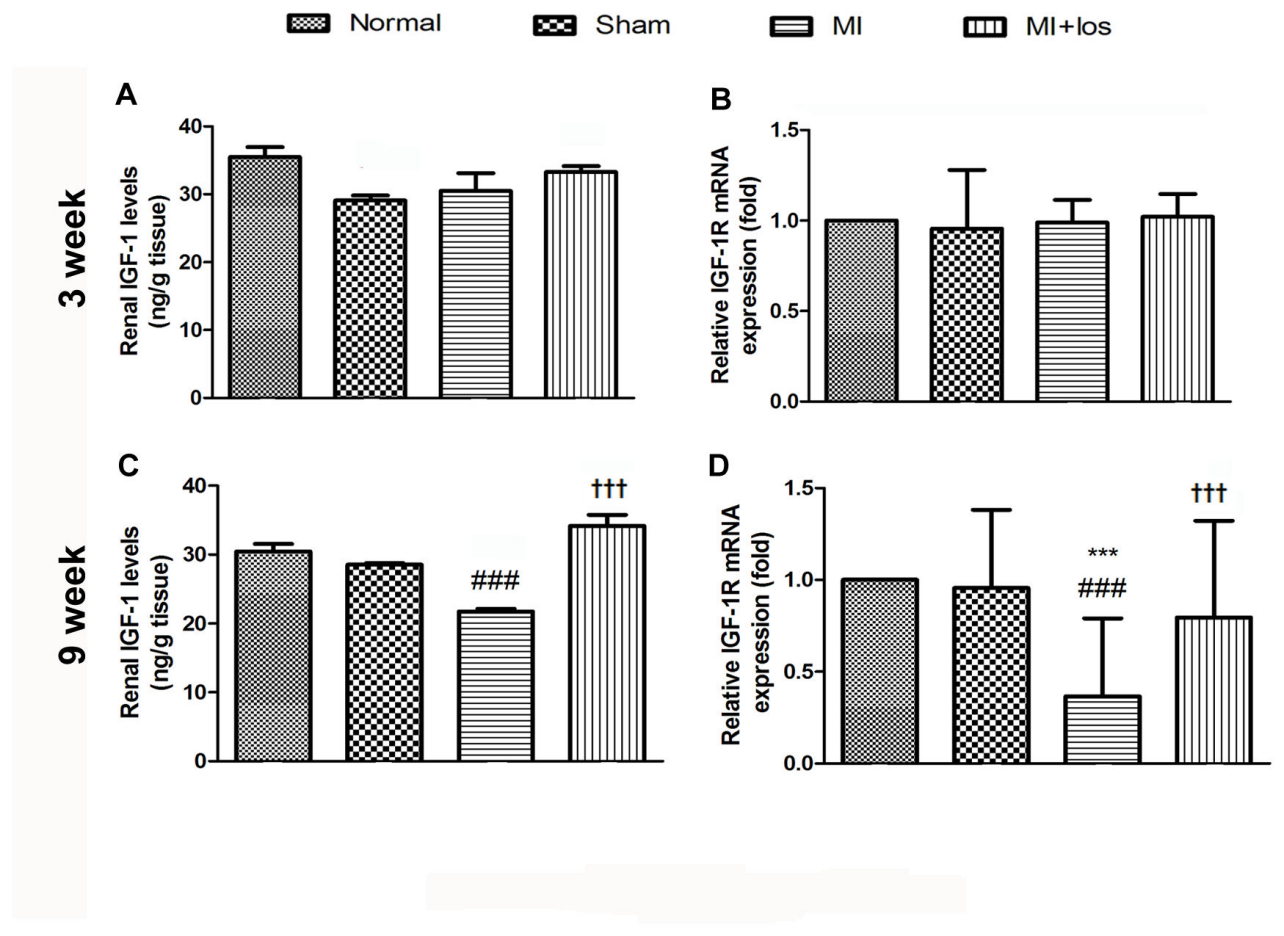
>Local changes of IGF-1 with its receptor IGF-1R in normal, sham, MI and MI+los rats. (A) Renal levels of IGF-1 at 3 weeks. (B) IGF-1R mRNA expression at 3 weeks. (C) Renal levels of IGF-1 at 9 weeks. (D) IGF-1R mRNA expression at 9 weeks. Data are presented as the mean ± SEM. ###*P* < 0.008 vs. normal; ***P < 0.008 vs. sham; ††† *P* < 0.008 vs. MI. IGF-1, insulin-like growth factor-1; MI, myocardial infarction; los, losartan.

In the present study, we examined 8-OHdG, a marker of oxidative stress, by immunostaining the glomerulus, including podocytes, and found that the MI-induced group had higher levels of 8-OHdG in the glomerulus when compared to the normal group at 3 (Figure 4A-B) and 9 (Figure 4D-E) weeks. Losartan treatment significantly prevented increased 8-OHdG-positive podocytes in the glomeruli of MI-induced animals. We also determined whether the IGF-1/IGF-1R/Akt pathway was involved in the ARB-mediated renoprotection in MI animal. Our findings revealed that IGF-1 levels decreased in renal cortical tissue 3 and 9 weeks after an operation of MI, whereas significant differences were only found at 9 weeks (Figure 5). MI rats treated with losartan had higher levels of IGF-1 in their renal cortical tissues than MI animals alone. Additionally, losartan treatment significantly restored down-regulated expressions of both IGF-1R mRNA (Figure 5) and protein as well as *p*-Akt protein expression after 9 weeks in MI animals (Figure 4F).

## Discussion

The present study revealed findings consistent with prior works that revealed progressive renal damage in MI patients [8] or MI models [18], and renal impairment as marked by the depletion and injury of podocytes and accompanied by increased blood cystatin C levels. The pathophysiology in cardiorenal syndrome is poorly understood and likely involves interrelated concepts such as low cardiac output, increased venous congestion and renal venous pressure, neurohormonal and inflammatory activation, and local changes [2,19]. In good agreement with other observations [1,8,9], the present study has shown that common renal damage after MI could be significantly prevented by RAS intervention, such as with the use of losartan. Furthermore, increased inflammatory cell infiltration and fibrosis within the kidneys occurring post-MI heart failure has also been demonstrated by our previous study [6] and the other study [10]. Our previous study [6] also demonstrated that increased inflammatory cell infiltration and fibrosis within the kidneys post-MI could be significantly attenuated by losartan treatment. Consequently, it can be speculated that enhanced RAS activation following MI may play a pivotal role in the cardiorenal interaction.

It has been shown that local synthesis is the major source of the AngII found in the kidneys [5,11]. Renal activation of AT1R by enhanced AngII seems to be independent of systemic RAS activation and is maintained even when the systemic vascular AT1Rs are effectively blocked [10,20]. The present study demonstrates that augmentation of AngII, coupled with its receptors, increased significantly in renal cortical tissue following MI. Consistent with other findings [11], the gene expression of AGT, but not renin mRNA, increased significantly following MI, and this increase was prevented by losartan administration. It is likely that the augmentation of AGT expression in renal tissue mediates local AngII production, contributing to the pathogenesis of progressive renal impairment in cardiorenal syndrome by activating renal AT1Rs.

Ample evidence supports the hypothesis that podocyte injury could be induced by the unduly activated intrarenal RAS axis, leading to functional disturbance of the glomerular filtration barrier. Podocytes not only respond to AngII through the activation of AT1Rs but are further capable of directly producing intrarenal AngII because of their possession of a local RAS [12,21]. Augmented activation of AngII binding to its receptor AT1R presented in podocytes will induce phenotype shifts, from being dynamically stable to adaptively migratory, causing podocyte depletion and focal segmental glomerulosclerosis [12-14,22]. Under normal physiological conditions, podocytes also play a specific role in the maintenance of intraglomerular RAS balance [23]. Selective overexpression of the AT1R in podocytes *in vivo* results in protein leakage and structural podocyte damage, while inhibition of RAS may prevent many deleterious processes in podocytes, thereby reducing the extent of injury [12]. However, little is known about the interaction between the local RAS and podocyte injury in MI subjects. Our current study reveals that enhanced activation of tissue-based RAS components was associated with podocyte injury as manifested by decreased glomerular staining of WT-1, a marker of podocytes, increased glomerular staining of desmin, and a decreased expression of podocin mRNA in rats with post-MI heart failure. We also showed that MI significantly induced the up-regulation of p16^ink4a^, a key mediator of cell cycle inhibitor for stress and aberrant signaling induced senescence, in glomerular podocytes. MI rats treated with losartan restored the expression of podocin and suppressed desmin- and p16^ink4a^-positive cells within the glomerulus. Our findings further support the concept that MI-induced activation of the local RAS is essentially involved in the onset and progression of podocyte injury.

Our findings also demonstrate that intrarenal activation of the RAS may stimulate local oxidative stress, contributing to significant glomerular podocyte injury, as revealed in other heart failure models [11]. Primary cardiac failure leads to stimulation of the RAS, potentiating progressive renal impairment through AngII-induced oxidative injury by reactive oxygen species generation [24]. Our findings demonstrate that podocyte injury and senescence are related to an up-regulated 8-OHdG in rats after MI, and losartan attenuates these abnormalities accompanied by a down-regulated 8-OHdG. These findings suggest that ARBs suppress the vicious cycle of RAS and oxidative stress in the kidney, resulting in glomerular podocyte protection.

The present study also indicates that podocyte injury and senescence are associated with a down-regulated IGF-1/IGF-1R/Akt pathway, while losartan could restore the activation of IGF-1/IGF-1R/Akt pathway and reduce podocyte injury. IGF-I and IGF-1R gene expressions are localized in glomerular podocytes, thus the alteration of IGF-1/IGF-1R signaling may play a detrimental role in maintaining the integrity of the podocyte [25,26]. IGF-1 activating its receptor IGF-1R may counteract AngII-mediated cellular senescence and oxidative stress in an AT1R-dependent manner [27]. Further studies have also shown that IGF-1 could prevent podocyte apoptosis via the activation of the phosphatidylinositol 3'-kinase pathway [28] and that AngII-decreased Akt phosphorylation could cause podocyte injury [12]. It has been shown that the local expression of IGF-I decreased significantly in animals post-MI heart failure [29], which occurring within the kidneys may be strongly correlated with severity of renal impairment [30]. Intervention of RAS with captopril increased the IGF-IR mRNA and protein expressions in myocardium post-MI [31]. Our present study may firstly reveal that local expression of IGF-1/IGF-1R signaling decreased significantly within the kidneys post-MI and losartan may restore the expressions of these abnormalities. However, our study demonstrates that changes in the IGF-1/IGF-1R/Akt signaling pathway occur at 9 weeks but not at 3 weeks post-MI. It may be hypothesized that IGF-1/IGF-1R/Akt pathway plays roles in protecting podocytes in a time-dependent after heart failure. Oxidative stress and inflammatory reaction rather than the inhibited IGF-1/IGF-1R/Akt signaling may be important detriments for the renal injury within 3 weeks. However, future experiments should investigate the sequential activation of the IGF-1/IGF-1R/Akt signaling pathway in AngII-induced podocyte senescence and injury.

## Conclusions

MI may induce activation of the local RAS coupled with renal impairment, as identified by podocyte injury in the glomerulus with resultant increased blood cystatin C levels. Cystatin C may be firstly explored as early marker for parenchymal damage of glomerular podocyte post-MI in the present study, thus further studies are needed to clarify their association. The therapeutic choice of losartan could attenuate renal impairment by inhibiting the AngII/AT1R-mediated cascade reaction post-MI. Inhibited glomerular oxidative stress and an up-regulated IGF-1/IGF-1R/Akt pathway may be important modulators involved in the process of podocyte protection in ARB treatment, but further studies with relevant pharmacological agents may help elucidate the mechanisms. Local RAS and potential mechanisms were tested in renal cortical tissues rather than in the glomerulus alone, which represent important limitations of the present study. Reduced blood pressure may discount the renoprotection of losartan, and the reduced renal damage in the present study may also be a consequence of improved cardiac function by losartan. However, whether there is a renoprotective role of ARBs in heart failure that is dependent of direct preservation of ventricular function has not been established. Losartan preferentially blocked the AT1R and thus much of the beneficial effects of losartan may be mediated by unopposed AngII on the AT2R, which may be the underlying mechanism in the present study. Therefore, genetically modified animal models with podocyte-specific deletions or overexpressed AngII receptors in conjunction with non-hypotensive doses of losartan or another pressure reducing agent as the control should be used to further test these hypotheses in future studies.

## Supporting Information

Table S1 Biological parameters of renal function.
