# Supplementary material for: Angiotensin II Receptor Blocker Attenuates Intrarenal Renin-Angiotensin-System and Podocyte Injury in Rats with Myocardial Infarction
Source: PLoS One. 2013 Jun 14;8(6):e67242. doi: 10.1371/journal.pone.0067242 (PMC3682995; doi:10.1371/journal.pone.0067242)
Supplement: Table S1 [file pone.0067242.s001.doc]

| **Table S1. Biological parameters of renal function** | | | | |
| --- | --- | --- | --- | --- |
|  | Normal | Sham | MI | MI+los |
| 3 week (n) | 8 | 8 | 14 | 20 |
| Urine protein (mg/d) | 2.8±0.28 | 3.1±0.14 | 5.2±0.03 | 4.4±0.45 |
| Serum creatinine (μmmol/L) | 67±2.90 | 76±3.25 | 80±7.24 | 67±2.39 |
| Blood urea nitrogen ( mmol/L ) | 4.4±0.28 | 5.6±0.21 | 6.3±0.19 ^a^ | 6.2±0.31 ^a^ |
| Blood cystatin C (mg/L) | 1.91±0.02 | 1.98±0.02 | 3.30±0.02 ^a b^ | 2.71±0.06 ^abc^ |
| 9 week (n) | 8 | 10 | 10 | 18 |
| Urine protein (mg/d) | 2.6±0.35 | 4.0±0.25 | 4.4±0.54 | 3.1±0.28 |
| Serum creatinine (μmmol/L) | 75±2.37 | 72±3.16 | 72±1.36 | 73±2.00 |
| Blood urea nitrogen ( mmol/L ) | 5.5±0.10 | 6.1±0.28 | 5.8±0.25 | 6.5±0.40 |
| Blood cystatin C (mg/L) | 2.09±0.04 | 2.06±0.06 | 3.63±0.06 ^a b^ | 2.88±0.07 ^abc^ |
| Data are presented as means ± SEM. *P*-value based on one-way ANOVA followed by LSD test. ^a^*P* < 0.008 vs. normal group; ^b^*P* < 0.008 vs. sham group; ^c^*P* < 0.008 vs. MI alone. MI, myocardial infarction; los, losartan. | | | | |
